# Evaluation of the Frequency of Hypokalemia in Patients on Diuretic Therapy for Heart Failure

**DOI:** 10.7759/cureus.91021

**Published:** 2025-08-26

**Authors:** Hafiza Aqsa Qaisar, Hussain Khan, Atiq Ul Rehman, Fnu Abdullah

**Affiliations:** 1 Internal Medicine, Ameer-ud-Din Medical College, Lahore, PAK; 2 Molecular Medicine and Pathology, University of the Punjab, Lahore, PAK; 3 Pathology, Federal Postgraduate Medical Institute (PGMI), Lahore, PAK; 4 Internal Medicine, University of Health Sciences, Lahore, PAK

**Keywords:** arrhythmias, cardiac, diuretics, heart failure, hypokalemia, potassium

## Abstract

Background: Hypokalemia is one of the frequent and clinically important electrolyte disturbances in patients with heart failure (HF), especially among patients under diuretic treatment. The objective of this study was to evaluate the occurrence of hypokalemia and determine clinical predictors of this condition in patients with HF hospitalized on diuretics.

Methods: A cross-sectional study was conducted on 150 patients with HF in a hospital. Patients who were taking loop or thiazide diuretics for at least one week were included. The participants were divided into the hypokalemic (K < 3.5 mmol/L, n = 47) and normokalemic (K ≥ 3.5 mmol/L, n = 103) groups, based on serum potassium. The demographics, comorbidities, type and dose of diuretic, and other clinical variables were also collected. An automated ion-selective electrode analyzer was utilized to determine the concentration of potassium. SPSS 26.0 (IBM Corp., Armonk, NY, US) was used for data analysis with the p-value < 0.05 as significant.

Results: The prevalence of hypokalemia in the study population was 47 (31.3%). It strongly correlated with the use of loop diuretics (34 (72.3%) vs. 54 (52.4%), p = 0.042), high-dose diuretics (23 (48.9%) vs. 30 (29.1%), p = 0.015), and the presence of diabetes mellitus (18 (38.3%) vs. 23 (22.3%), p = 0.028). Loop diuretic use (odds ratio (OR) = 2.10, p = 0.023) and its high dose (OR = 1.80, p = 0.018) were the independent predictors of hypokalemia by logistic regression analysis.

Conclusion: The study demonstrated that hypokalemia was common among HF patients receiving diuretic therapy, particularly loop diuretics in high doses. Although loop diuretic use and higher doses were significant independent predictors, the efficacy of interventions like dose adjustment or potassium-sparing drugs needs to be further validated in future randomized controlled trials.

## Introduction

Hypokalemia is a serum potassium concentration of <3.5 mmol/L and is a common electrolyte imbalance in heart failure (HF) patients and individuals treated with diuretics [1]. Loop diuretics, in HF, block the renal NKCC2 transporter in the thick ascending limb of Henle’s loop, contributing to greater urinary potassium and magnesium loss, increasing the likelihood of hypokalemia, neuromuscular response, arrhythmia, and sudden cardiac death [2].

Hypokalemia is common in patients with HF, with an incidence of 2% to 10%, depending on the population, diuretic dose, and HF subtype [3]. In Japan, registry data showed that 3.4% of discharged patients had hypokalemia associated with thiazide use and older age [4]. In contrast, more than a third of older patients starting loop diuretics needed potassium supplementation, showing a clinically important prescribing cascade [5]. The clinical burden of hypokalemia is independently associated with increased mortality, HF hospitalization, and cardiovascular events, particularly in chronic HF with comorbid chronic kidney disease (CKD) [6]. Furthermore, research indicates hypokalemia and hypomagnesemia may predispose to falls, weakness, and arrhythmic potential in older HF populations [7].

Despite the adverse effects, there is decreased knowledge on ideal monitoring, diuretic effects on potassium status, and methods to reduce risks. Modern recommendations suggest close electrolyte monitoring when initiating or adjusting diuretics or adding mineralocorticoid receptor antagonists (MRAs) to assist in maintaining potassium homeostasis [8]. This highlights a substantial requirement for evidence-based clinical guidelines, which may individualize surveillance and therapy intervention to lower HF-associated complications and mortality.

The objective of this study was to determine the prevalence of HF patients on diuretics with hypokalemia. The purpose was also to assess independent predictors and the type and dose of diuretic. Furthermore, it aimed to determine evidence-based surveillance to avoid hypokalemia-induced morbidity during HF care.

## Materials and methods

The purpose of the study was to determine the prevalence and predictors of hypokalemia in patients with HF on diuretic therapy in this prospective observational study. It was carried out in the Department of Cardiology at Punjab University affiliated with Federal Postgraduate Medical Institute (PGMI), Lahore, from October 2023 to April 2024. The study was ethically approved by the Institutional Review Board (Ref: 143-01-2023), and written informed consent was obtained from all participants before enrolment.

A non-probability consecutive sampling strategy was used to recruit 150 adult patients. OpenEpi version 3.0.0 (released 2013, Atlanta, GA, US) was used to calculate the sample size based on a 30% prevalence of hypokalemia among HF patients, 95% confidence level, 80% power, and a margin of error of 7.5% [9]. The single proportion formula was applied, where Z = 1.96, p = 0.30, and d = 0.075, which yielded a minimum sample size of 150 patients. Patients (aged 18 years and above) with HF (both reduced and preserved ejection fraction) and taking loop or thiazide diuretics for at least one week were included in the study. Patients with CKD stage 5; on dialysis; using potassium-sparing agents or supplements; hospitalized within the past 14 days; with endocrine disease (adrenal/thyroid), active infection, cancer, or hepatic disease; receiving potassium-manipulating medications; or non-compliant with the study protocol were excluded. All patients with a confirmed diagnosis of HF who met the inclusion criteria and presented to the cardiology department during the study period were approached and recruited after obtaining informed consent. No eligible patient who gave consent was excluded from the recruitment.

The participants were divided into two groups based on serum potassium concentrations: hypokalemia (K < 3.5 mmol/L, n = 47) and without hypokalemia (K ≥ 3.5 mmol/L, n = 103). The concentration of serum potassium was determined by an automated indirect ion-selective electrode (ISE) analyzer. Information regarding demographics, comorbidities (e.g., diabetes), nature of diuretics and doses, and concomitant medication was obtained. Diuretic dose was categorized into the standard and high-dose groups. High-dose loop diuretics were ≥40 mg/day furosemide (or equivalent: ≥20 mg/day torsemide or ≥1 mg/day bumetanide). A high dose of thiazide diuretics was defined as ≥50 mg/day of hydrochlorothiazide or equivalent (e.g., ≥25 mg/day chlorthalidone or ≥2.5 mg/day indapamide). Patients with doses lower than these thresholds were labeled as standard-dose. Compliance with diuretic treatment was established through review of prescriptions and an interview.

Data were analyzed with SPSS version 26.0 (released 2019, IBM Corp., Armonk, NY, US). The chi-squared test was used to evaluate the categorical variables, and the independent t-test was used to determine the continuous variables. Independent predictors of hypokalemia were calculated using multivariable logistic regression. A p-value < 0.05 was taken as statistically significant.

## Results

The current study evaluated 150 patients with HF taking diuretics to identify hypokalemia, prevalence, and its predictors. Hypokalemia (<3.5 mmol /L) was observed in 47 patients (47 (31.3%)). Hypokalemia was significantly linked to loop diuretics and increased doses (p = 0.042 and p = 0.015, respectively). Diabetes mellitus was higher among hypokalemic patients (p = 0.028), but it was not an independent predictor. Logistic regression supported the use of loop diuretics and high doses as key predictors, which shows the importance of active potassium monitoring. Table 1 shows the comparison of demographic and clinical characteristics between the two groups.

**Table 1:** Demographic and clinical characteristics in hypokalemic and normokalemic patients with heart failure n: number of participants; SD: standard deviation; %: percentage *Significance at p-value < 0.05

Parameter	Hypokalemia (n = 47)	No hypokalemia (n = 103)	Test used	Test value	Significance (p-value)
Mean age (years), mean ± SD	62.4 ± 11.5	60.2 ± 12.8	Independent t-test	t = 1.08	0.282
Male (%)	26 (55.3%)	60 (58.3%)	Chi-squared test	χ² = 0.12	0.728
Diabetes mellitus (%)	18 (38.3%)	23 (22.3%)	Chi-squared test	χ² = 4.82	0.028*
Hypertension (%)	29 (61.7%)	60 (58.3%)	Chi-squared test	χ² = 0.17	0.680

Both groups had a similar age and gender distribution, with a mean age of 62.4 and 60.2, respectively (p = 0.282). However, diabetes mellitus was more frequent in the hypokalemia group (18 (38.3%) vs. 23 (22.3%), p = 0.028), which suggests an association between electrolyte imbalances and diabetes mellitus. The association of diuretic dosage and type with potassium concentrations in HF is presented in Table 2.

**Table 2:** Diuretic dosage and type n: number of participants; %: percentage *Significance at p-value < 0.05

Parameter	Hypokalemia (n = 47)	No hypokalemia (n = 103)	Test used	Test value	Significance (p-value)
Loop diuretics (%)	34 (72.3%)	54 (52.4%)	Chi-squared test	χ² = 5.07	0.042*
Thiazide diuretics (%)	13 (27.7%)	49 (47.6%)	Chi-squared test	χ² = 5.07	0.042*
High-dose diuretics (%)	23 (48.9%)	30 (29.1%)	Chi-squared test	χ² = 6.04	0.015*

The use analysis of diuretics observed that hypokalemia occurred more frequently when loop diuretics were used compared to thiazide diuretics (54 (72.3%) vs. 34 (27.7%), p = 0.042). Furthermore, there was a significantly higher risk associated with hypokalemia among the patients who took diuretics in higher doses (23 (48.91%) vs. 30 (29.12%), p = 0.015). These findings highlight the significance of dosage or type of diuretic in regulating potassium levels. Table 3 indicates the predictors of hypokalemia among HF patients.

**Table 3:** Multivariate regression analysis of predictors of hypokalemia n: number of participants; OR: odds ratio; CI: confidence interval *Significance at p-value < 0.05

Predictor	Adjusted OR	95% CI	Test used	Test value	Significance (p-value)
Loop diuretic use	2.10	1.10–4.00	Logistic regression (Wald test)	5.18	0.023*
High-dose diuretics	1.80	1.10–3.20	Logistic regression (Wald test)	5.59	0.018*
Diabetes mellitus	1.45	0.85–2.48	Logistic regression (Wald test)	1.85	0.174
Age (per year increase)	1.01	0.98–1.04	Logistic regression (Wald test)	0.45	0.501

In logistic regression, loop diuretics (odds ratio (OR) = 2.10, 95% confidence interval (CI) = 1.10-4.00, p = 0.023) and increased diuretic doses (OR = 1.80, 95% CI = 1.10-3.20, p = 0.018) were independent factors contributing to hypokalemia. Diabetes mellitus and age were not significant predictors after adjustment. This reinforces the importance of medication management in the prevention of hypokalemia among HF patients.

## Discussion

The purpose of this study was to determine the prevalence and clinical predictors of hypokalemia among patients with HF receiving diuretics. Hypokalemia was found to be 31.3% prevalent with loop diuretics and higher dosing as statistically significant independent predictors. These results correspond with a study, demonstrating a range of prevalence rates of hypokalemia among HF patients of 3%-50%, with prevalence variation depending on the level of treatment and clinical center [10]. A meta-analysis study also concluded that the intense dosing of loop diuretics was associated with increased risk of hypokalemia and negative cardiovascular events [11]. Meanwhile, a hospital-based cohort discovered that increased loop diuretic doses indicated hypokalemia despite chronological age and kidney performance adjustment [12].

These observations are supported by the identified predictors in this study. In contrast, a small number of other studies indicated the potentially greater predictive value of demographic factors such as sex or baseline potassium, which the current study failed to confirm, perhaps due to hospital-based sampling and strict inclusion criteria [13]. However, the findings showed no differences in sex-related outcomes and were consistent with an evaluation of HF registry data, which indicated heterogeneity based on cohort characteristics [14].

The relationship between hypokalemia and arrhythmias has been established, and potassium deficiencies have been associated with slow cardiac repolarization and electrical instability [15]. Coexisting hypomagnesemia has also been demonstrated to exacerbate the presence of neuromuscular symptoms and the likelihood of falling in both geriatric HF populations [16]. These mechanisms are supported by current study findings, as the rates of hypokalemia were higher among patients with comorbid diabetes and those on high-dose diuretics. MRAs, including finerenone, have been found protective due to their action against loss of potassium and have shown positive effects on clinical outcomes [17,18]. Another analysis also supported the use of MRAs due to their potassium-sparing effects in HF. This confirms the approach of initiating MRA early in patients using loop diuretics [19]. This study has implications related to the significance of planned potassium observation and personalized diuretics. Complications can be minimized by improving MRA use or potassium supplementation in high-risk patients.

This study has several strengths. It addressed a clinically important question in a high-risk HF population, generating strong clinical utility. Internal validity was enhanced through standardization of potassium, performed via an automated ISE analyzer and strict inclusion/exclusion criteria. The multivariable logistic regression enhanced the analysis by determining independent predictors, beyond simple relationships. Lastly, results were structured and presented in a way that improves clarity and interpretation.

The limitations of the present study include a single-center and cross-sectional design that may potentially impact the generalizability of results. Employing a non-probability consecutive sampling method led to selection bias, since the sample would not be fully representative of the wider population of HF patients on diuretics. This may also influence the external validity and generalizability of our results. As this was a cross-sectional study, only associations were identified; causality between diuretic use and hypokalemia could not be inferred. Other confounding factors, including dietary potassium, renal function differences, and concomitant drugs, were not completely taken into consideration. Moreover, baseline serum potassium concentrations and stratification by CKD stages (other than stage 5) were not controlled, which may have affected the results. To further develop electrolyte management strategies, studies are recommended to employ large multicentric studies, prospective populations, and randomized trials in the future.

## Conclusions

The prevalence of hypokalemia in this study was 31.3% in patients with HF taking diuretics, but it was much higher in patients taking loop diuretics and higher doses. These two variables were significant independent predictors using logistic regression; however, other factors, including age, gender, and diabetes mellitus, were non-significant after adjustment. The results highlight the need for close potassium monitoring and consideration of potassium-sparing interventions, but further prospective and randomized controlled studies are required to determine whether they can improve clinical outcomes.

## References

[1] Surendran K, Joseph BM, Vilapurathu JK (2022). Treatment strategies of hypokalemia in heart failure. J Ind Coll Cardiol.

[2] Alexander RT, Dimke H (2024). Molecular mechanisms of loop diuretics on renal calcium and magnesium transport. Acta Physiol (Oxf).

[3] Bushahu H, Wajanga B, Kidenya B, Nkandala I (2024). Prevalence and factors associated with potassium abnormalities among outpatients with heart failure taking diuretics in a tertiary referral hospital in Tanzania. East Afr Health Res J.

[4] Miura Y, Higuchi S, Kohno T (2022). Association of potassium level at discharge with long-term mortality in hospitalized patients with heart failure. J Clin Med.

[5] Wang GH, Morris EJ, Smith SM, Hallas J, Vouri SM (2023). Continued potassium supplementation use following loop diuretic discontinuation in older adults: an evaluation of a prescribing cascade relic. J Am Geriatr Soc.

[6] Tafesse E, Hurst M, Sugrue D (2022). Serum potassium as a predictor of adverse clinical outcomes in patients with increasing comorbidity burden. Eur Heart J Qual Care Clin Outcomes.

[7] van Poelgeest EP, Handoko ML, Muller M, van der Velde N (2023). Diuretics, SGLT2 inhibitors and falls in older heart failure patients: to prescribe or to deprescribe? A clinical review. Eur Geriatr Med.

[8] Wu L, Rodriguez M, El Hachem K, Krittanawong C (2024). Diuretic treatment in heart failure: a practical guide for clinicians. J Clin Med.

[9] Bhardwaj R, Agrawal U, Vashist P, Manna S (2024). Determination of sample size for various study designs in medical research: a practical primer. J Family Med Prim Care.

[10] Krogager ML, Kragholm K, Thomassen JQ (2021). Update on management of hypokalaemia and goals for the lower potassium level in patients with cardiovascular disease: a review in collaboration with the European Society of Cardiology Working Group on Cardiovascular Pharmacotherapy. Eur Heart J Cardiovasc Pharmacother.

[11] Kapelios CJ, Bonou Μ, Malliaras K (2022). Association of loop diuretics use and dose with outcomes in outpatients with heart failure: a systematic review and meta-analysis of observational studies involving 96,959 patients. Heart Fail Rev.

[12] Patel RB, Fonarow GC, Greene SJ (2021). Kidney function and outcomes in patients hospitalized with heart failure. J Am Coll Cardiol.

[13] Wouda RD, Boekholdt SM, Khaw KT (2022). Sex-specific associations between potassium intake, blood pressure, and cardiovascular outcomes: the EPIC-Norfolk study. Eur Heart J.

[14] Otaki Y, Watanabe T, Yamaguchi R (2023). Hypokalemia, kidney function, and clinical outcomes in heart failure with preserved ejection fraction. Circ J.

[15] Lloyd C, Mohar C, Priano J (2022). Hypokalemic cardiac arrest: narrative review of case reports and current state of science. J Emerg Nurs.

[16] Negru AG, Pastorcici A, Crisan S, Cismaru G, Popescu FG, Luca CT (2022). The role of hypomagnesemia in cardiac arrhythmias: a clinical perspective. Biomedicines.

[17] Vardeny O, Vaduganathan M, Claggett BL (2025). Finerenone, serum potassium, and clinical outcomes in heart failure with mildly reduced or preserved ejection fraction. JAMA Cardiol.

[18] Yu W, Luo F, Rao J (2025). Adverse effects of finerenone in patients with heart failure: a systematic review and meta-analysis. Front Cardiovasc Med.

[19] Masi S, Dalpiaz H, Piludu S, Piani F, Fiorini G, Borghi C (2025). New strategies for the treatment of hyperkalemia. Eur J Intern Med.

